# MedFusionGAN: multimodal medical image fusion using an unsupervised deep generative adversarial network

**DOI:** 10.1186/s12880-023-01160-w

**Published:** 2023-12-07

**Authors:** Mojtaba Safari, Ali Fatemi, Louis Archambault

**Affiliations:** 1 Département de Physique, de génie Physique et d’Optique, et Centre de Recherche sur le Cancer, Université Laval, Québec City, QC Canada; 2 Service de Physique Médicale et Radioprotection, Centre Intégré de Cancérologie, CHU de Québec - Université Laval et Centre de recherche du CHU de Québec, Québec City, QC Canada; 3 Department of Physics, Jackson State University, Jackson, MS USA; 4 Department of Radiation Oncology, Gamma Knife Center, Merit Health Central, Jackson, MS USA

**Keywords:** IGART, Deep learning, MRI, Brain tumor

## Abstract

**Purpose:**

This study proposed an end-to-end unsupervised medical fusion generative adversarial network, MedFusionGAN, to fuse computed tomography (CT) and high-resolution isotropic 3D T1-Gd Magnetic resonance imaging (MRI) image sequences to generate an image with CT bone structure and MRI soft tissue contrast to improve target delineation and to reduce the radiotherapy planning time.

**Methods:**

We used a publicly available multicenter medical dataset (GLIS-RT, 230 patients) from the Cancer Imaging Archive. To improve the models generalization, we consider different imaging protocols and patients with various brain tumor types, including metastases. The proposed MedFusionGAN consisted of one generator network and one discriminator network trained in an adversarial scenario. Content, style, and L1 losses were used for training the generator to preserve the texture and structure information of the MRI and CT images.

**Results:**

The MedFusionGAN successfully generates fused images with MRI soft-tissue and CT bone contrast. The results of the MedFusionGAN were quantitatively and qualitatively compared with seven traditional and eight deep learning (DL) state-of-the-art methods. Qualitatively, our method fused the source images with the highest spatial resolution without adding the image artifacts. We reported nine quantitative metrics to quantify the preservation of structural similarity, contrast, distortion level, and image edges in fused images. Our method outperformed both traditional and DL methods on six out of nine metrics. And it got the second performance rank for three and two quantitative metrics when compared with traditional and DL methods, respectively. To compare soft-tissue contrast, intensity profile along tumor and tumor contours of the fusion methods were evaluated. MedFusionGAN provides a more consistent, better intensity profile, and a better segmentation performance.

**Conclusions:**

The proposed end-to-end unsupervised method successfully fused MRI and CT images. The fused image could improve targets and OARs delineation, which is an important aspect of radiotherapy treatment planning.

**Supplementary Information:**

The online version contains supplementary material available at 10.1186/s12880-023-01160-w.

## Introduction

Magnetic resonance imaging (MRI) and computed tomography (CT) provide complementary information about the human body, both anatomical and physiological. For instance, the former could acquire high-resolution soft-tissue contrast anatomical and functional images from nuclear spin, and the latter provides geometrically corrected images of electron density. The electron density is used by treatment planning systems to calculate heterogeneous dose distribution. However, CT with a limited soft-tissue contrast makes the region of interests (ROIs) and organs at risk (OARs) delineations more difficult than MRI, which has a superior soft-tissue contrast. However, unlike CT images, MRI is lacking electron density information and requires particular MRI sequences with short echo time to visualize bony anatomy.

While image fusion approaches were initially investigated to improve image quality and combine information for better diagnosis [1], they have since then been proposed for many applications from surgical guidance to reduce data storage volume [2]. Nevertheless, multi-modal image fusion has yet to be widely integrated into routine clinical use [3]. This can be attributed in part to the highly technical nature of the fusion process. Furthermore, because of recent increases in computing power, most clinical software can now seamlessly navigate between image datasets, limiting the need for image fusion. With the rapid rise of deep learning (DL) for image processing, image fusion is seeing an increased interest in non-medical and medical images [4].

In radiotherapy, multi-modal image fusion is crucial in aiding target delineation as an integral part of treatment planning [5]. For instance, in brachytherapy, the fusion of MRI and CT scans has reduced the maximum dose to healthy organs at risk [6]. However, the simultaneous use of two or more medical images or their side-by-side comparison can introduce the potential for human errors and impose increased computational demands.

Most clinically employed multi-modal image fusion methods are limited to rigid registration, followed by manual switching between image datasets or a straightforward overlay of two images (e.g., superimposing a semi-transparent color representation of a positron emission tomography scan onto a grayscale CT image). In recent years, radiation therapy has seen a notable surge in its reliance on imaging for treatment planning and daily patient monitoring, notably through a technique known as image-guided adaptive radiation therapy (IGART) [7]. However, IGART tends to be more time-consuming than traditional radiotherapy, particularly when MRI is utilized [8]. IGART mandates the evaluation of daily images before each treatment fraction to determine whether adjustments to the treatment plan are necessary. Therefore, there is a pressing need for advanced image fusion methods to seamlessly combine multi-modal images into a unified representation, optimizing the information available to the clinical team during daily IGART procedures. Such enhancements can boost treatment efficiency and patient throughput while reducing the risk of human error.

Furthermore, certain intracranial stereotactic radiosurgery (SRS) platforms, such as the Gamma Knife (GK), rely exclusively on MRI for treatment planning. In this context, fused images can enhance treatment accuracy by improving glioma delineation [9] and refining dose calculations beyond the current simplistic tissue maximum ratio approach.

The image fusion process generally seeks to produce a new image from multiple images that satisfy the following criteria: (a) the fused image must retain the information of the source images, (b) redundant information must be discarded and (c) the fused image must be free of image artifact and noise (either initially present or added by the fusion process) [10].

To fulfill these requirements, we are proposing a novel fusion method to combine high-resolution 3D T1-Gd MRI and CT images using an end-to-end unsupervised medical fusion generative adversarial network (GAN), MedFusionGAN, that balance the MRI soft tissue contrast and CT bone and electron density information. Typically, GANs consist of a generator ($\mathcal {G}$) network and a discriminator ($\mathcal {D}$) network. While $\mathcal {G}$ attempts to combine the MRI soft tissue with CT bone and electron density data, $\mathcal {D}$ is trained to distinguish between the source images and the fused image guiding $\mathcal {G}$ to maximize the information of both source images in the fused image.

The MedFusionGAN employed a *patchGAN* discriminator [11] in the fusion process of the MRI and CT images. The significant contribution of MedFusionGAN are highlighted as follows:

- the network was trained under an unsupervised framework.
- only one discriminator was used to fuse source images and therefore can be extended to fuse more than two source images.
- both CT and MRI source images were used in training $\mathcal {D}$.
- perceptual loss [12], gradient loss, and structural similarity index (SSIM) [13] loss were used in combination to preserve texture and structure of the source images.

The goal of this work is (1) to develop a novel image fusion method to combine MRI and CT images in a way that maximize the information content in the fused image and (2) to compare this method with other fusion techniques using a wide array of quality metrics. We believe that access to high quality image fusion could improve and facilitate structure delineation in radiation therapy and thus help workflows such as IGART that rely on large volume of images.

The rest of this paper is organized as follows: Related work describes related work on the image fusion task. Material details the dataset and pre-processing steps. Method presents the proposed GAN, including network architecture and loss functions. Results illustrates the visual and quantitative results and compared to four state-of-the-art traditional fusion methods. Finally, Discussion and Conclusion discuss the significance of this new technique and its possible use in the context of IGART and GK.

## Related work

### Traditional method for image fusion

Traditional fusion methods can be categorized as spatial domain and transform domain techniques. The former involves fusing the source images at the pixel level while the latter performs the fusion in an intermediate domain called transformed domain [13, 14]. Spatial domain methods, including high-pass filter, principal component analysis, and independent component analysis have been applied to fuse visible and infrared images [15]. However, spatial domain methods fuse images with high spectral and spatial distortions [16].

Transformed domain approaches perform the fusion in a non-spatial domain (e.g. frequency domain) and thus require to transform the image before applying the fusion. For instance, Diwakar *et al.* applied non-subsampled shearlet transform to calculate the low and high frequency components of the source images that were combined using engineered filters [17]. Also, the images in the images in the non-spatial domain were combined using clustering algorithm to improve the fusion images’ contrast and content [18, 19]. Although these techniques are robust to distortions, they generate a noisy fusion image. In addition, the transform domain approaches use a similar transformation for the source images. Still, different semantics may cause an unnecessary amount of redundant information in the fused image [4].

### CNNs for image fusion

Convolutional neural networks (CNNs) are composed of kernels whose values are learned through the error back-propagation [20]. CNNs have been widely used in medical image processing such as segmentation, classification, registration, and computer vision like satellite images fusion [21–23].

In the context of image fusion, CNNs are used in different scenarios including pixel-wise weighting map extraction, feature extraction, and feature extraction with reconstruction. The first method extracts pixel-wise weighting factors that are used for different images [24]. The second method extracts image features and, then, hand-crafted methods are implemented to combine them [25]. The third approach is an end-to-end method that extracts and combines the features to fuse the images [26]. For instance, Zhang *et al.* proposed a network comprising two branches to extract features from source images where they were combined in different levels [27]. Similarly, Li *et al.* trained a network with two branches [28], however, a transformer module was used to extract local and global features.

Even though the fusion objectives are clear (i.e. maximizing the information and minimizing the noise and artifacts), there is generally no *ground truth*. The absence of a ground truth implies that image fusion should be considered as an unsupervised learning task. This complicates the use of several well-established CNN architectures for image processing such as the U-Net [29] because these have been mainly studied for supervised learning tasks.

### GANs for image fusion

GANs are widely used in medical imaging such as synthetic image generation, registration, and image reconstruction [30, 31]. A GAN is an implicit technique that typically consists of two networks; a generator ($\mathcal {G}$) and a discriminator ($\mathcal {D}$). $\mathcal {G}$ produces data with distribution $P_G$ to be as close as possible to the distribution of the real data $P_{data}$ while $\mathcal {D}$ is trained to distinguish between the true data and generated samples [32] (see Fig. 3b). Both the $\mathcal {G}$ and the $\mathcal {D}$ are trained in this adversarial game framework to ultimately generate the realistic data. GAN was first defined as:

1 $$\begin{aligned} \underset{G}{\min }\ \,\underset{D}{\max }\,\, V(G, D) = \mathbb {E}_{\textbf{x} \sim P_{data}(\textbf{x})}\log \left( D(\textbf{x}) \right) + \mathbb {E}_{\mathbf {\textbf{z}} \sim P_{\textbf{z}}(\mathbf {\textbf{z}})}\left[ \log \left( 1- D(G(\textbf{z})) \right) \right] \end{aligned}$$

GANs outperformed most explicit and implicit approaches in different domains such as in generating realistic images and image-to-image translation [33, 34].

#### GAN based image fusion

Satellite images fusion methods were proposed using GANs. For instance, *FusionGAN* fused the visible and infrared images [35]. The visible images with texture information and infrared images with thermal radiation were used to train the $\mathcal {G}$ while only infrared images were used to train $\mathcal {D}$. Thus, the *FusionGAN* aimed to preserve visible image texture and infrared thermal radiation. The $\mathcal {D}$ attempted to make the fused image indistinguishable from infrared image.

In order to leverage the visible and infrared images in training the discriminator, *DDcGAN* proposed a GAN with two $\mathcal {D}$ [36], one $\mathcal {D}$ for each source image. However, it required a careful training to prevent mode collapse [36] (i.e., generating data similar to only one of the source images).

## Material

### Dataset

We used a publicly available multicenter medical GLIS-RT dataset from the Cancer Imaging Archive [37] consisting 230 patients (100 males and 130 females). All patients with different brain tumor types underwent 3D T1-Gd, T2-fluid-attenuated inversion recovery MRI sequences, and a CT scan under different imaging protocols to improve the generalization [38] of the MedFusionGAN. The brain tumor types were glioblastoma (GBM - 198 cases), anaplastic astrocytoma (AAC - 23 cases), astrocytoma (AC - 5 cases), anaplastic oligodendroglioma (AODG - 2 cases), and oligodendroglioma (ODG - 2 case). We used $80\%$ (11246 image slices) for taining and $20\%$ of data (2276 image slices) for testing our method that were not used in train step. There was no overlap between the training and testing datasets.

The median of the CT and 3D T1-Gd images’ resolution was $0.66 \times 0.66 \times 2.5$ mm$^3$ (standard deviation $0.09 \times 0.09 \times 0.12$ mm$^3$) and $0.94 \times 0.94 \times 1.$ mm$^3$ (standard deviation $0.24 \times 0.24 \times 1.21$ mm$^3$), respectively. The MRI imaging parameters were (median $\pm$ std); TE = $2.98 \pm 3.86$ ms, TR = $2200 \pm 1031.76$ ms, TI = $900 \pm 235.50$ ms, and flip angle = $9.0 \pm 5.45 ^\circ$. About $30\%$ of data were acquired using MRI scanners with B$_0$ of 1.5T and the others were acquired using 3T scanners. Of 230 cases, 55 cases were obtained using GE MRI scanners and the rests were obtained using Siemens MRI scanners.

### Dataset preparation

**Fig. 1:**
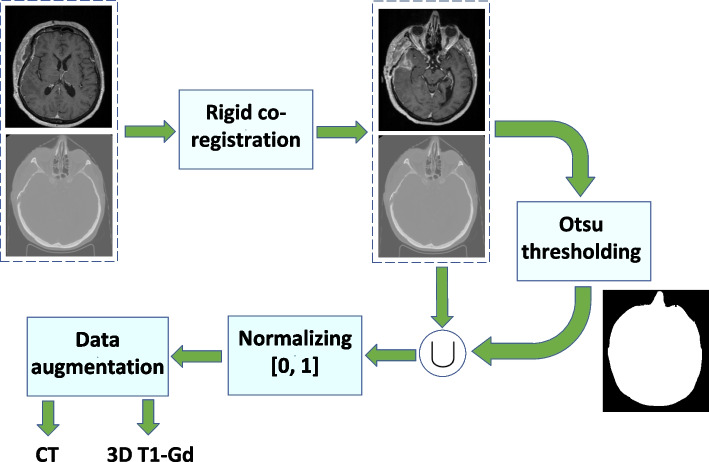
After co-registering CT and MRI, the bed and tabletop were removed using Otsu method, and the data were normalized between 0 and 1. Finally, the data augmentation methods were employed to improve the network generalization

**Fig. 2:**
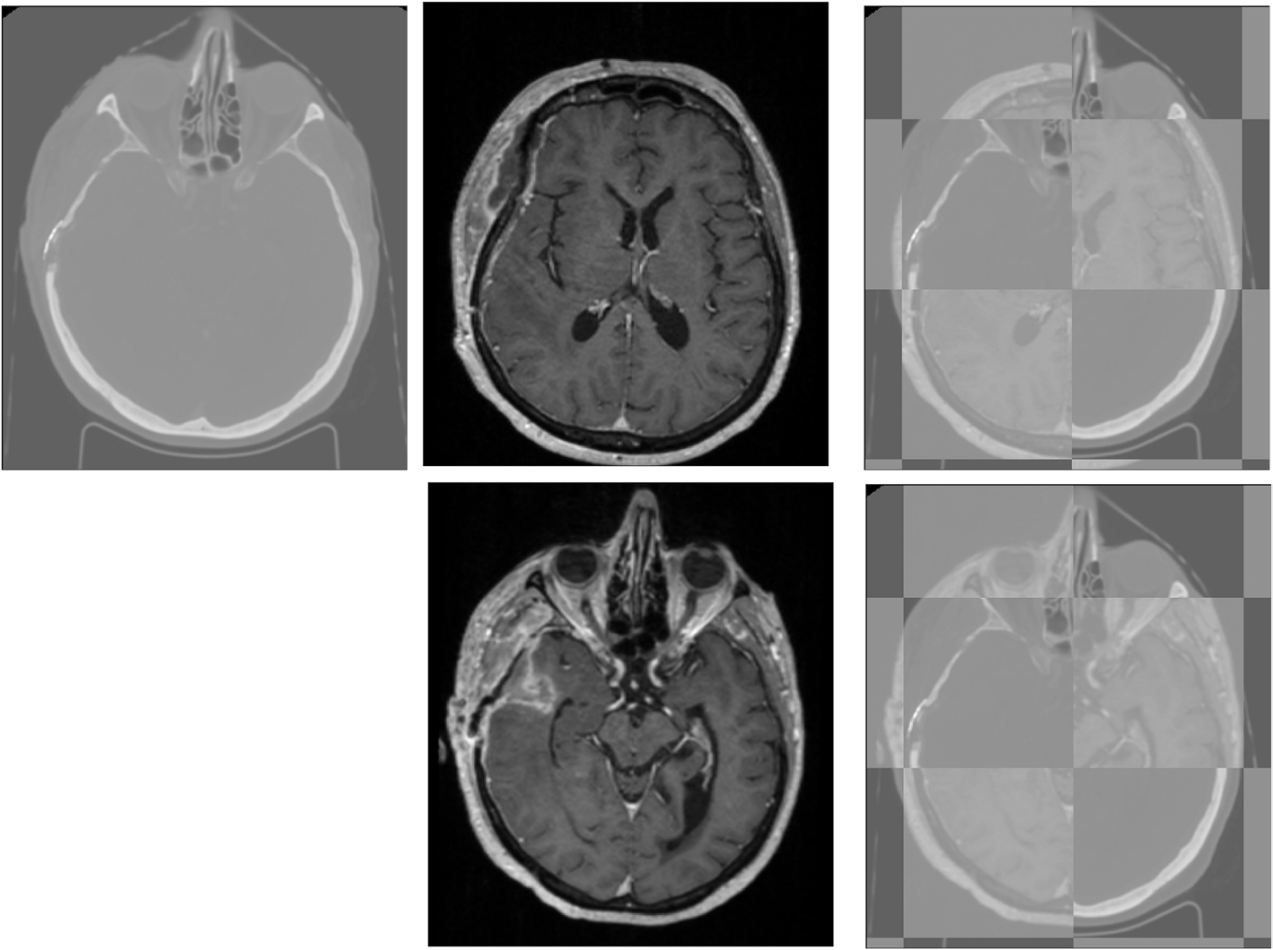
The first row from left to right shows the CT image (fixed), 3D T1-Gd (moving image), and checkerboard plots of the input images before co-registration. The second row from left to right illustrates the 3D T1-Gd after co-registration and the checkerboard plots of the input images after co-registration

Figure 1 illustrates the preprocessing steps applied to CT and 3D T1-Gd images that were as follows:

1. Rigid co-registration (using FSL FLIRT^1^) to spatially transfer the MRI onto the CT [39]. Normalized mutual information with 128 histogram bins was used as a similarity measure [40] (see Fig. 2 for an example of the rigid co-registration).
2. Binary mask extraction using the Otsu method [41] for both source images. Comparison of those masks was used to remove the bed and tabletop in the CT images
3. The final masks generated using a closing morphological operator to remove the holes inside the masks.
4. Image intensities normalization between 0 and 1 as given in (2).
  2 $$\begin{aligned} y_{normalized} = \frac{x - x_{\min }}{x_{\max } - x_{\min }} \end{aligned}$$
  where $x_{\max }$ and $x_{\min }$ denote the maximum and minimum pixel values in a given image slice.
5. The following data augmentation methods [42] were used;
  Horizontal and vertical flips, random rotation of up to $20^\circ$, and random grid distortion all with probability of $30\%$.

## Method

**Fig. 3:**
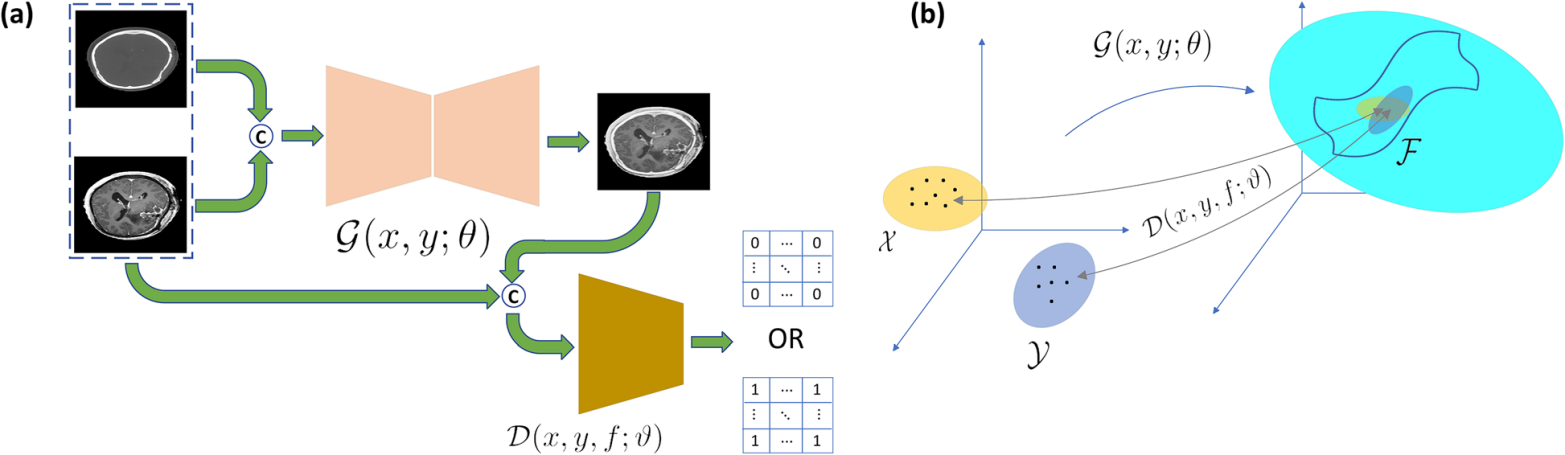
The MedFusionGAN framework $\mathcal {G_\theta }:\ \{\mathcal {X}, \mathcal {Y}\} \rightarrow \mathcal {F}$. The $\mathcal {G}_\theta$ aims to map the CT ($\mathcal {X}$) and MRI ($\mathcal {Y}$) images data distribution under the unsupervised framework to image fusion ($\mathcal {F}$) while the $\mathcal {D}_\vartheta$ quantify the data distribution distance between the source images and the fusion image (**a**). The fusion images will have a probability distribution sampled from source images (**b**)

GANs are implicit generative models that, in the context of image fusion, learn a generator $\mathcal {G}_\theta$ to map the MRI ($\mathcal {X}$) and CT ($\mathcal {Y}$) images data manifolds to the fusion image data manifold $\mathcal {F}$ ($\mathcal {G}_\theta : \{\mathcal {X}, \mathcal {Y}\} \rightarrow \mathcal {F}$ where $\mathcal {X}$, $\mathcal {Y}$, and $\mathcal {F}$ are MRI, CT, and fusion images data space) [43] (see Fig. 3a). At the same time, the $\mathcal {D}_\vartheta$ estimates the distance between the data distribution of the source images and the fused image leading $\mathcal {G}_\theta$ to share data distribution of the source images and not only one of them (see Fig. 3b).

The GAN training involves two steps. First, updating the $\mathcal {D}$ as given in (3).

3 $$\begin{aligned} \mathcal {L}_{adv}(G,D)&= \mathbb {E}_{x\sim p_{data}(I_{mri}), y\sim p_{data}(I_{ct})}\left[ \log D([x, y]) \right] \nonumber \\&\quad +\mathbb {E}_{x\sim p_{data}(I_{mri}), y\sim p_{data}(I_{ct})} \left[ \log \left( 1 - \lambda _1^D D([G(x, y), x]) \right. \right. \nonumber \\&\quad \left. \left. - \lambda _2^D D([G(x, y), y])\right) \right] \end{aligned}$$

where $\lambda _1^D + \lambda _2^D = 1$ we used $\lambda _1^D = \lambda _2^D = 0.5$, and the $\left[ \bullet \right]$ is a concatenation operator. By adapting the *Patch*GAN discriminator with double-channel input, it was possible to work on local image patches. The local patches with size $M\times M$, which *M* was smaller than image size, were used instead of whole image to discriminate the source images from fusion. The discriminator output averaged over all patches was taken as the final output of $\mathcal {D}$ [11]. Hence, it will improve the spatial resolution of the fusion images.

In the image fusion context, the goal of the generator is to preserve appearance and texture information of the source images with the aim of minimizing the loss $\mathcal {L}(G) = \mathcal {L}_{1}(G) + \mathcal {L}_{content}(G)$. Therefore, beside the $\mathcal {L}_1$ loss between source images and fusion image, three content losses were used as given in Eq. (4) to preserve texture and structure of the source images.

4 $$\begin{aligned} \mathcal {L}_{content}(G) = \lambda _1^G \mathcal {L}_{gradient}^{(I_f, I_{mri})} + \lambda _2^G \mathcal {L}_{SSIM}^{(I_f, I_{mri})} + \lambda _3^G \mathcal {L}_{perceptual}^{(I_f, I_{ct})} \end{aligned}$$

where $\mathcal {L}_{gradient}^{(I_f, I_{mri})}$ and $\mathcal {L}_{SSIM}^{(I_f, I_{mri})}$ were the gradient and the SSIM [44] losses between “fused” and “mri” images, respectively, are given in (5) and (6). The $\mathcal {L}_{gradient}^{(I_f, I_{mri})}$ loss minimized the difference between the MRI and fusion images edge information. Thus, MRI edges information (soft tissue contrast) was delivered to the fused images.

5 $$\begin{aligned} \mathcal {L}_{gradient}^{(I_f, I_{mri})} = {\mathbb {E} \left[ | {\nabla I_F - \nabla I_{mri}} | \right] } \end{aligned}$$

The SSIM loss (6) was used to constrain structural similarity between fused and MRI source image.

6 $$\begin{aligned} \mathcal {L}_{SSIM}^{(I_f, I_{mri})} = 1 - SSIM(I_f, I_{mri}) \end{aligned}$$

where $SSIM(I_f, I_{mri})$, SSIM similarity metric, is defined as follows

7 $$\begin{aligned} SSIM(I_f, I_{mri}) = \frac{\left( 2 \mu _{mri} \mu _f + C_1\right) \left( 2 \sigma _{mri,f} + C_2\right) }{ \left( \mu _{mri} ^ 2 + \mu _f ^ 2 + C_1\right) \left( \sigma _{mri} ^ 2 + \sigma _f ^ 2 + C_2\right) } \end{aligned}$$

$\mu _{mri}$, $\sigma _f$, and $\sigma _{mri,f}$ are the local mean, local standard deviation, and local covariance between MRI and Fused images, respectively. $C_1$ and $C_2$ are the constant parameters to stabilize the SSIM.

The pre-trained VGG16 network was used to estimate the perceptual loss [45] between CT and fuse image. The perceptual loss preserves CT bone structure and texture. Because VGG16 had been trained on a RGB dataset, the CT and fusion images were repeated three times along the channel before calculating the loss. Equation (8) gives the mathematical formulation of the perceptual loss.

8 $$\begin{aligned} \mathcal {L}_{perceptual}^{(I_f, I_{ct})} = \mathbb {E} \left[ \Vert \phi (I_f) - \phi (I_{ct}) \Vert _2^2\right] \end{aligned}$$

where $\phi (\bullet )$ is the pretrained VGG16 network.

Finally, the GAN objective to fuse source images (MRI and CT) can be described as follows:

9 $$\begin{aligned} G^* = \arg \,\,\underset{G}{\min }\ \,\, \underset{D}{\max }\,\,\, \mathcal {L}_{adv}(G,D) + \mathcal {L}(G) \end{aligned}$$

The generator consisted of nine convolution blocks in the down-sampling and up-sampling blocks. The down-sampling block was inspired by the ResNet block [46] that consists of convolution layers followed by batch normalization, a Leaky ReLU activation function with negative slope of 0.2, and a skip connection. The up-sampling block comprised an up-sampling layer and two similar ResNet blocks.

We used the Adam optimizer with learning rate of $2\times 10^{-4}$. The batch size and the epoch number were 8 and 40, respectively. The MedFusionGAN training time was 116.9 minutes using NVIDIA RTX 3090 GPUs.

The proposed method was implemented using the PyTorch 2 framework and ran on a workstation equipped with two NVIDIA RTX 3090 GPUs.

## Results

Multi-modal images CT and high-resolution MRI images with complementary information are required to deliver prescribed dose to the targets and spare the OARs. However, working with several images will adversely affect radiation therapy time and increase computation burden of the treatment planning system.

The proposed method was qualitatively and quantitatively compared with 15 state-of-the-art methods, eight of these methods uses deep learning (DL) while the others are traditional. The traditional and DL methods are listed in Table 1.

**Table 1:** Traditional and DL methods are listed with their corresponding reference number and code URL that was provided by the authors

Methods	Name	Reference	Code URL
Traditional	directional discrete cosine transform and principal component analysis (DDCT-PCA)	[14]	https://www.mathworks.com/matlabcentral/fileexchange/46169-directional-discrete-cosine-transform-and-principal-component-analysis-based-image-fusion
	hybrid multi-scale decomposition (HMSD)	[13]	https://github.com/bitzhouzq/Hybrid-MSD-Fusion
	fourth-order partial differential equations (FPDE)	[15]	https://www.mathworks.com/matlabcentral/fileexchange/63570-multi-sensor-image-fusion-based-on-fourth-order-partial-differential-equations
	gradient transfer fusion (GTF)	[47]	https://github.com/jiayi-ma/GTF
	multi-exposure image fusion (MEF)	[48]	https://github.com/tkd1088/multi-exposure-image-fusion
	infrared and visible image fusion (IVF)	[49]	https://github.com/LYJ903118120/IVFusion
	multi-focus images fusion (GFDF)	[50]	https://github.com/bitname/Multi-focus-image-fusion-GFDF
Deep learning	CNN-Fuse	[51]	https://github.com/budaoxiaowanzi/image-fusion
	CU-Net	[52]	https://github.com/cindydeng1991/TPAMI-CU-Net
	SESF-Fuse	[53]	https://github.com/Keep-Passion/SESF-Fuse
	DSAGAN	[54]	https://github.com/jeffsonfu/DSAGAN
	U2Fusion	[55]	https://github.com/hanna-xu/U2Fusion
	SwinFusion	[56]	https://github.com/Linfeng-Tang/SwinFusion
	IFCNN	[57]	https://github.com/uzeful/IFCNN
	FusionGAN	[35]	https://github.com/jiayi-ma/FusionGAN

The quantitative and qualitative comparisons between the MedFusionGAN and traditional and DL methods are presented in different subsections to facilitate the comparisons.

### Qualitative comparisons

Although a fusion image target or ground truth is not available, acceptable fusion images must satisfy three criteria. First, they need to include information of (both) source images. Thus they need to contain MRI soft-tissue contrast and CT bone information. Second, they must not add spatial and spectral distortions to the fusion images, which is especially important for tumor dataset as they might be misinterpreted as tumors. Finally, they need to discard redundant information that are available in the source images.

#### Traditional methods

**Fig. 4:**
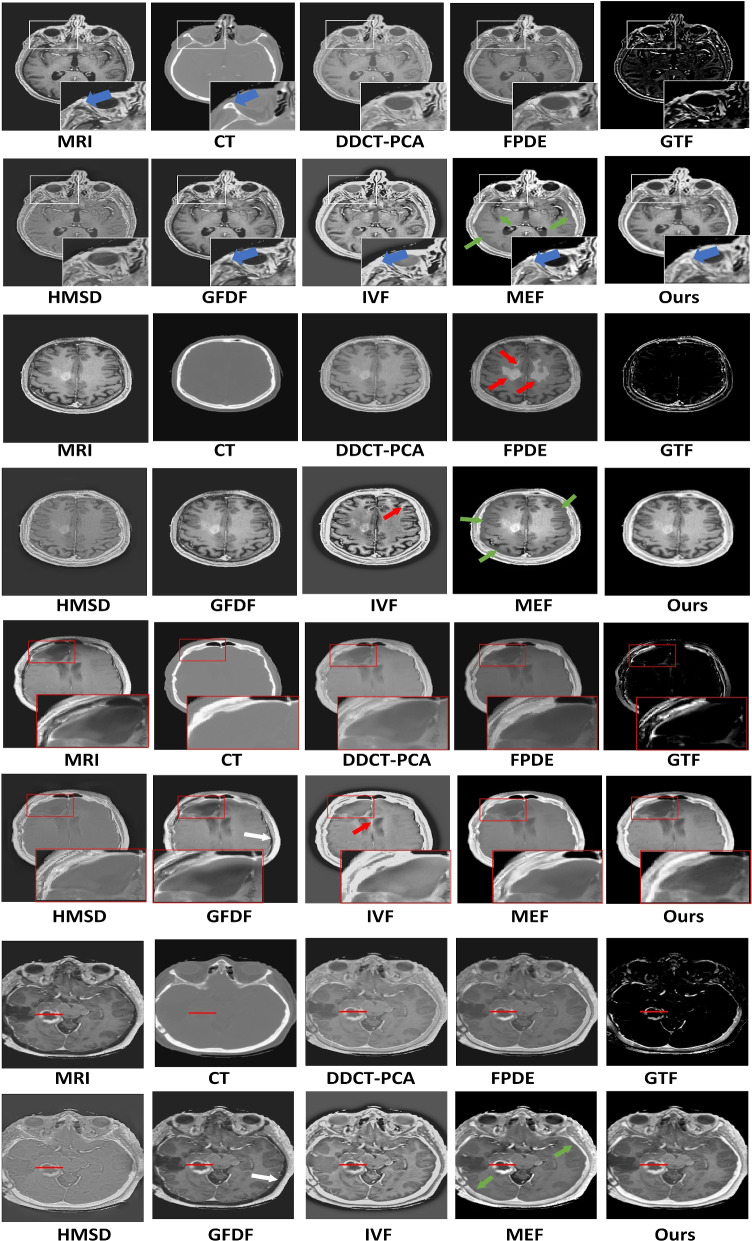
Qualitative comparison of the proposed MedFusionGAN with seven state-of-the-art traditional fusion methods are illustrated for four different image slices. Red and green arrows show the image artifacts. Blue arrows within the white boxes indicate the distinction between skull and scalp. Yellow arrow shows the indistinguishable border between white matter and gray matter. Red boxes are served to zoom in to the cancerous regions to show the image contrasts of different traditional fusion methods. Red horizontal lines indicate the location of the image profile shown in Fig. 8a

The MedFusionGAN could qualitatively generate fuse images with T1-Gd MRI soft tissue and CT bone contrasts shown in Fig. 4 without adding spatial and spectral distortions to the fused images. By comparison, the FPDE fusion method generated fused images with substantial spatial distortions as indicated by red arrows in Fig. 4. In addition, the GTF method did not fuse high-resolution MRI images and CT images with a large dynamic range difference where the skulls with very high signal intensity in CT masked out the MRI soft tissue signals. GFDG could barely transfer bone information of CT images to the fusion images (white and blue arrows in Fig. 4). Although IVF could combine the MRI soft-tissue contrast and CT bone information, it increased the intensity of MRI soft-tissue as the scalp in MRI had a similar intensity as CT bone indicated by blue arrows in Fig. 4 within white boxes. The MedFusionGAN generated the fusion images with more visible boundary between bone and scalp as zoomed in the white boxes. IVF blurred the boundary between the white matter and the gray matter (see yellow arrow Fig. 4). MEF preserved the intensity of soft tissues of MRI, but it add a thick band of spatial distortion between the brain and skull and increased thickness of the gray matter. MEF distortions are indicated by green arrows in Fig. 4.

Although the DDCT-PCA, HMSD, IVF, and MEF fusion methods neither add spatial distortions nor unsuccessful in delivering MRI soft-tissue contrast and CT bone information, they generated fusion images with lower soft-tissue contrasts compared with the MedFusionGAN (see red boxes in Fig. 4). We assumed the MRI delivered the ideal soft-tissue contrasts compared with the CT images. Hence, the proposed method could deliver excellent soft-tissue contrasts as it is very close to the MRI.

#### Deep learning methods

**Fig. 5:**
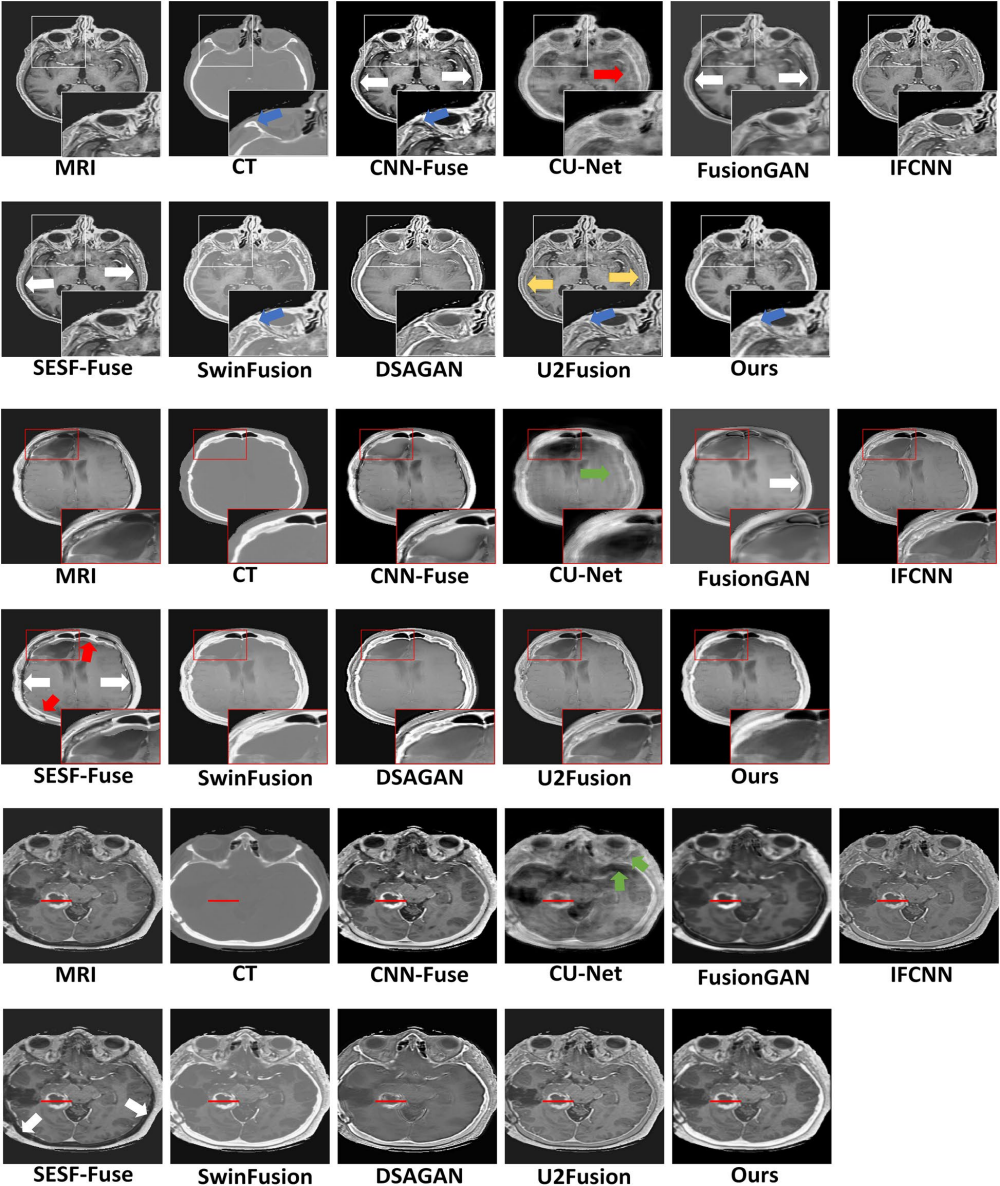
Qualitative comparison of the proposed MedFusionGAN with eight state-of-the-art DL fusion methods are illustrated for three different image slices. Green arrows show the spatial distortion similar to the MRI motion artifacts. Blue arrows within the white boxes indicate the distinction between skull and scalp. Red boxes are served to zoom in to the cancerous regions to show the image contrasts of different traditional fusion methods. White arrows and yellow arrow indicate the regions that the CT bone information was not and with low spatial contrast delivered to the fusion images, respectively. Red horizontal lines indicate the location of the image profile shown in Fig. 8b

Similarly, the MedFusionGAN could successfully combine MRI and CT images without adding distortions to the fusion images (see Fig. 5). However, FusionGAN and SESF-Fuse methods did not deliver CT bone information to the fusion images and CNN-Fuse partially delivered it (white arrows in Fig. 5). Also, CT bone information was delivered with low spatial contrast by the U2Fusion method (yellow arrow in Fig. 5). FusionGAN, CU-Net, and DSAGAN generated fusion images with low soft-tissue contrast compared with the MedFusionGAN.

CU-Net added spatial distortions to the fusion images that were similar to the MRI motion artifact and gradient-induced phase error (green arrows in Fig. 5). Similarly, SESF-Fuse added spatial distortions to the fusion images at the boundary between brain and skull indicated by red arrows in Fig. 5.

Although SwinFusion did not add spatial distortion to the generated fusion images, the MedFusionGAN generated fusion images with better spatial contrast around the cancerous region as illustrated by red boxes in Fig. 5 and better contrast between skull and scalp illustrated by blue arrows within white boxes.

### Quantitative comparisons

Nine quantitative measures were reported to evaluate image fusion results. These measures were: entropy (ENT), standard deviation (STD), mean gradient (MG), spatial frequency (SF), mutual information (MI), normalized cross-correlation (NCC), peak signal-to-noise ratio (PSNR), $Q^{XY/F}$ [58], and SSIM [44]. The quantitative metrics are explained in the Supplementary document.

#### Traditional methods

**Fig. 6:**
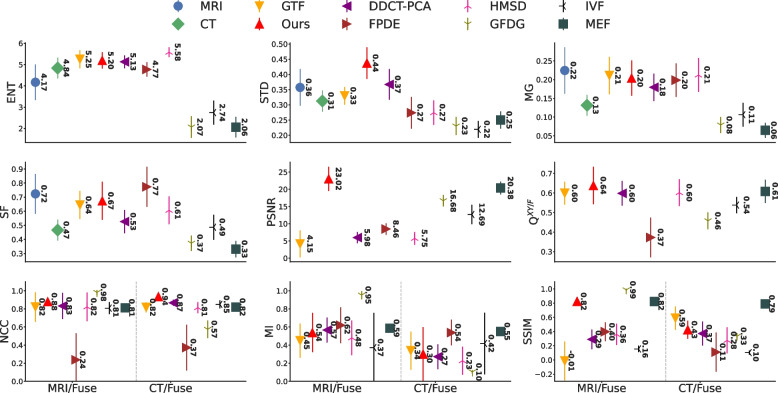
Quantitative metrics (mean $\pm$ std) of our proposed GAN method, MedFusionGAN, and the seven traditional methods are illustrated. *Abbreviations:* ENT, entropy; STD, standard deviation; PSNR, peak signal-to-noise ratio; MG, mean gradient; SF, spatial frequency; NCC, normalized cross-correlation; MI, mutual information; SSIM, structural similarity index

Considering that the GTF method did not preserve soft tissue contrast, a noticeable amount of image artifacts from FPDE when fused with the CT and 3D T1-Gd MRI image sequences, and missing CT bone information from GFDF (see Fig. 4), they will be excluded from the quantitative comparisons. However, their quantitative results are still presented in Fig. 6. In addition, we summarized the quantitative values (mean $\pm$ std) in Table 2 where an astrict ($*$) mark represents that the quantitative metric of the given fusion method was statistically insignificant different from our method (*p*-value $>0.05$).

**Table 2:** Mean±STD of our GAN method and other traditional methods are summarized. The GTF, FPDF, and GFDF will be excluded from the quantitative comparisons as they did not transfer MRI soft-tissue contrast, add a noticeable amount of spatial distortion, and did not transfer CT bone information, respectively. **Bold** indicates the best results. Underline indicate a better result than ours that was excluded because it did not satisfy the fusion criteria

Method	ENT	STD	PSNR	$Q^{XY/F}$	MG	SF	NCC	MI	SSIM
GTF	5.25$\pm$0.42	0.33$\pm$0.03	4.15$\pm$3.91	0.6$\pm$0.06	$\underline{0.21\!\pm \!0.05}$*	$\underline{0.64\!\pm \!0.1}$*	0.82$\pm$0.12	$\underline{0.39\!\pm \!0.21}$*	0.29$\pm$0.37
DDCT-PCA	5.13$\pm$0.3*	0.37$\pm$0.05	5.98$\pm$1.65	0.6$\pm$0.06	0.18$\pm$0.04	0.53$\pm$0.08*	0.85$\pm$0.11	0.42$\pm$0.2*	0.33$\pm$0.16
FPDE	4.77$\pm$0.34	0.27$\pm$0.05	8.46$\pm$1.65	0.37$\pm$0.10	$\underline{0.20\!\pm \!0.04}$*	0.77$\pm$0.14	0.3$\pm$0.28	0.58$\pm$0.18	0.25$\pm$0.26
HMSD	**5.58** $\varvec{\pm }$ **0.23**	0.27$\pm$0.04	5.75$\pm$1.86	0.6$\pm$0.07	**0.21**$\varvec{\pm }$0.05*	0.61$\pm$0.10	0.82$\pm$0.12	0.35$\pm$0.22	0.32$\pm$ 0.17
GFDF	2.07 ± 0.51	0.23 $\pm$ 0.03	16.68 $\pm$ 1.64	0.46 $\pm$ 0.04	0.08 $\pm$ 0.02	0.37 $\pm$ 0.06	0.78 $\pm$ 0.22	$\underline{0.53\!\pm \!0.43}$*	$\underline{0.66\!\pm \!0.33}$*
IVF	2.74± 0.57	0.22± 0.03	12.69 ± 2.79	0.54 ± 0.04	0.11 ± 0.03	0.49 ± 0.09	0.83 ± 0.06	0.4 ± 0.36*	0.13 ± 0.05
MEF	2.06± 0.48	0.25± 0.03	20.38± 1.86	0.61± 0.06	0.06± 0.02	0.33± 0.06	0.82± 0.04	**0.57** $\varvec{\pm }$ **0.04**	**0.81** $\varvec{\pm }$ **0.03**
Ours	5.2$\pm$0.38	**0.44** $\varvec{\pm }$ **0.05**	**23.02**$\varvec{\pm }$3.5	**0.64** $\varvec{\pm }$ **0.1**	**0.20** $\varvec{\pm }$ **0.05**	**0.67** $\varvec{\pm }$ **0.14**	**0.91** $\varvec{\pm }$ **0.04**	0.42$\pm$0.29	0.62$\pm$0.22

* is not statistically different (*p*-value $> 0.05$) from our proposed MedFusionGAN method.

*Abbreviations:* *ENT* entropy, *STD* standard deviation, *PSNR* peak signal-to-noise ratio, *MG* mean gradient, *SF* spatial frequency, *NCC* normalized cross-correlation, *MI* mutual information, *SSIM* structural similarity index

Our method fused the source images with the highest STD, PSNR, Q$^{XY /F}$, NCC, MG, and SF values, where the difference between our method and the other methods were statistically significant (see Table 2). The MedFusionGAN generated fusion images with the second highest ENT values after the HMSD method. In addition, our method along with two other methods (DDCT-PCA and IVF) produced fusion images with the second highest MI (since the difference between our method and other methods was statistically insignificant with *p*-value $> 0.05$). The MedFusionGAN generated fusion images with the second highest values of SSIM. These results demonstrate that our method generated images with better spatial contrast (highest STD), preserve the structural information of the source images (highest ENT, PSNR, and NCC), and produced the images with the highest amount of edge/gradient information (highest Q$^{XY /F}$. SF, and MG).

#### Deep learning methods

**Fig. 7:**
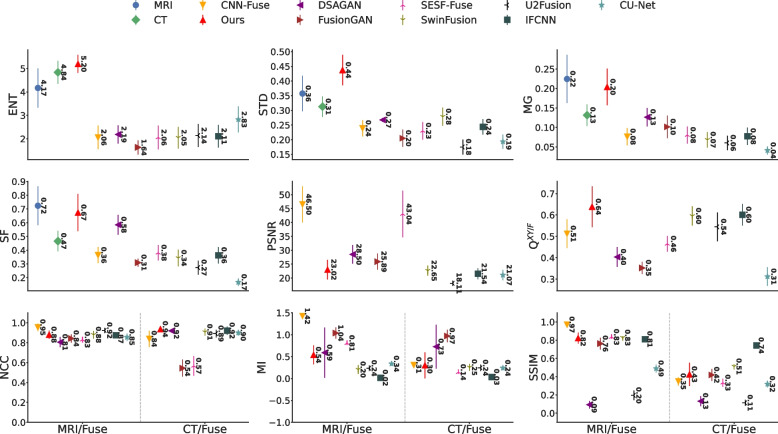
Quantitative metrics (mean $\pm$ std) of our proposed GAN method, MedFusionGAN, and other DL methods are illustrated. *Abbreviations:* ENT, entropy; STD, standard deviation; PSNR, peak signal-to-noise ratio; MG, mean gradient; SF, spatial frequency; NCC, normalized cross-correlation; MI, mutual information; SSIM, structural similarity index

CNN-Fuse, FusionGAN, and SESF-Fuse methods are excluded from quantitative comparisons because they generated fusion images without CT bone structure, which is in contradiction with the need to retain information of all source images [10] as listed in Qualitative comparisons section. However, their quantitative metrics are still shown in Fig. 7 and summarized in Table 3.

Our method generated fused images with the highest ENT, STD, Q$^{XY/F}$, MG, and SF metric values, where the difference between our method and the other DL methods was statistically significant. Both our method and U2Fusion method generated fusion images with the highest NCC with the statistically insignificant difference (*p*-value $> 0.05$).

**Table 3:** Mean±STD of our GAN method and the deep learning methods are summarized. CNN-Fuse, FusionGAN, and SESF-Fuse will be excluded from the quantitative comparisons as they did not generated fusion images that contain CT bone structures. **Bold** indicates the best results. Underline indicate a better result than ours that was excluded because it did not satisfy the fusion criteria

Method	ENT	STD	PSNR	$Q^{XY/F}$	MG	SF	NCC	MI	SSIM
CNN-Fuse	2.06± 0.5	0.24± 0.03	46.5± 6.49	0.51± 0.07	0.08± 0.02	0.36± 0.06	0.9± 0.09*	0.87± 0.56	0.66± 0.31
SESF-Fuse	2.06± 0.51	0.23± 0.03	43.04 ± 8.41	0.46± 0.04	0.08± 0.02	0.38± 0.06	0.7± 0.15	0.47± 0.33*	0.58± 0.25*
SwinFusion	2.05± 0.46	0.28± 0.03	22.65± 1.78*	0.6± 0.04	0.07± 0.02	0.34± 0.06	0.89± 0.04	0.23± 0.09	0.67± 0.16
IFCNN	2.11± 0.48	0.24± 0.03	21.54± 1.96	0.6± 0.05	0.08± 0.02	0.36± 0.06	0.9± 0.04	0.03± 0.05	**0.78**$\varvec{\pm }$**0.04**
U2Fusion	2.14± 0.49	0.18± 0.03	18.11± 0.95	0.54± 0.07	0.06± 0.02	0.27± 0.05	**0.91**$\varvec{\pm }$0.04*	0.24± 0.07	0.15± 0.06
DSAGAN	2.19± 0.39	0.27± 0.01	**28.5**$\varvec{\pm }$**3.33**	0.4± 0.05	0.13± 0.02	0.58± 0.07	0.86± 0.07	**0.66**$\varvec{\pm }$**0.54**	0.11± 0.05
CU-Net	2.83 ± 0.55	0.19 ± 0.02	21.07 ± 1.84	0.31 ± 0.04	0.04 ± 0.01	0.17 ± 0.03	0.88 ± 0.04	0.29 ± 0.07	0.4 ± 0.09
FusionGAN	1.64± 0.3	0.21± 0.03	25.89± 2.88	0.35± 0.03	0.1± 0.03	0.31± 0.03	0.69± 0.16	1.0± 0.14	0.59± 0.18
Ours	**5.2**$\varvec{\pm }$**0.38**	**0.44**$\varvec{\pm }$**0.05**	23.02$\pm$3.5	**0.64**$\varvec{\pm }$**0.1**	**0.20**$\varvec{\pm }$**0.05**	**0.67**$\varvec{\pm }$**0.14**	**0.91**$\varvec{\pm }$**0.04**	0.42$\pm$0.29	0.62$\pm$0.22

* is not statistically different (*p*-value $> 0.05$) from our proposed MedFusionGAN method

*Abbreviations:* *ENT* entropy, *STD* standard deviation, *PSNR* peak signal-to-noise ratio, *MG* mean gradient, *SF* spatial frequency, *NCC* normalized cross-correlation, *MI* mutual information, *SSIM* structural similarity index

Although CNN-Fuse, FusionGAN, and SESF-Fuse generated fusion images with higher PSNR than our method (underlined in Table 3), they did not contain CT bone information. Considering this, our method generated fusion images with the second highest PSNR and MI values.

#### Comparing ROIs

To determine the soft-tissue contrast of different fusion methods around tumors, the intensities along the tumor were plotted. Similar to the previous Traditional methods and Deep learning methods sections, the fusion methods that did not satisfy the fusion criteria were excluded from comparing the tumor contrast.

**Fig. 8:**
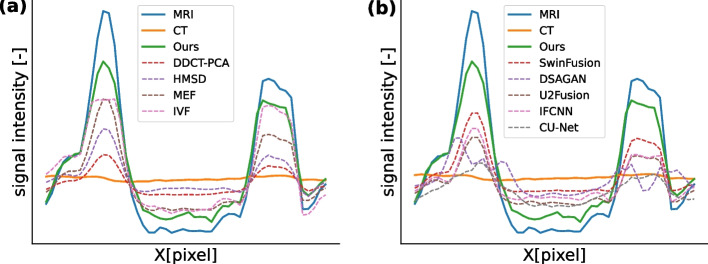
The image profiles are represented along the red lines shown on the last image slice of **a** Fig. 4 for traditional fusion methods and **b** Fig. 5 for DL fusion methods

Given the fact that a maximum peak to a minimum peak (peak-to-peak) of signal intensities at tumor boundaries indicates spatial contrast, MRI had the highest spatial contrast at both sides of the profile of the tumor intensity. The tumor instensity profiles are illustrated in Fig. 8a for traditional and Fig. 8b for DL methods. At right hand side of the tumor, IVF was slightly lower than our method and MEF had the third highest signal intensities (see Fig. 8a). The intensity profiles in Fig. 8a indicates that the MedFusionGAN provides consistent contrast (similar differences at both sides of the tumors).

**Fig. 9:**
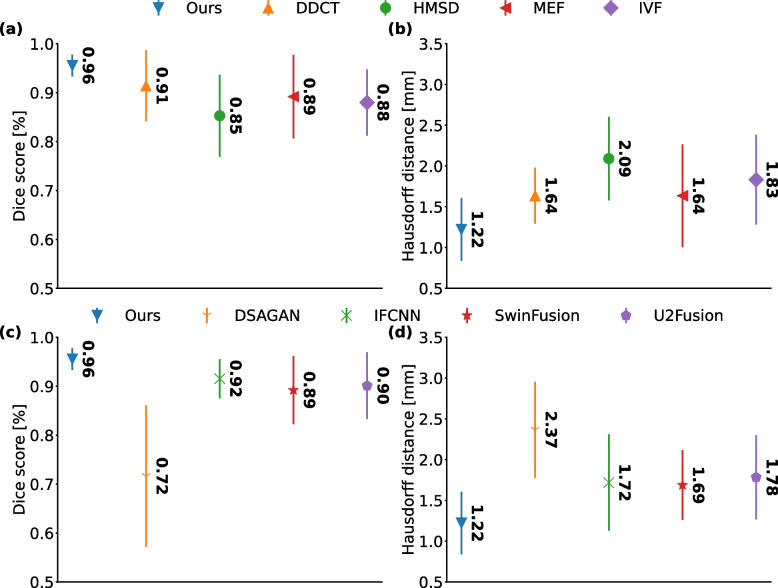
The quantitative metrics comparing brain tumor contours outlined on fusion images. Mean ± std values for traditional method are **a** Dice score metric, **b** HD metric, for deep learning algorithms are **c** Dice score metric, and **d** HD metric

To compare the fusion methods in segmenting the tumors, two segmentation metrics Sørensen Dice coefficient (Dice score) and Hausdorff distance (HD) metrics (mean $\pm$ std) were reported for the contours outlined on fusion images generated using traditional and DL algorithms except with GTF, FPDF, CNN-Fuse, FusionGAN and SESF-Fuse methods because they did not satisfy the fusion criteria (see Fig. 9). A semi-automatic level tracing method (using 3D Slicer 3) was employed to reduce humane bias in tumor contouring.

Dice score quantifying the segmentation similarity were $0.85 \pm 0.08$ for HMSD, $0.88 \pm 0.07$ for IVF, $0.89 \pm 0.09$ for MEF, and $0.91 \pm 0.07$ for DDCT-PCA method. It was significantly increased to $0.96 \pm 0.02$ (*p*-value $< 0.05$) for MedFusionGAN method (see Fig. 9a).

HD that calculate the maximum distance between the nearest points on the segmentation regions was calculated for the fusion methods. HD had a reverse trend as dice score where it was $2.09 \pm 0.51$ mm for HMSD, $1.83 \pm 1.10$ mm for IVF, $1.64 \pm 1.26$ mm for MEF, and $1.64 \pm 0.34$ mm for DDCT-PCA. MedFusionGAN with a lower HD of $1.22 \pm 0.38$ was significantly (*p*-value $< 0.05$) lower than the other fusion methods (see Fig. 9b).

Dice score values for different DL models are illustrated in Fig. 9c. The dice score values were $0.72 \pm 0.14$ for DSAGAN, $0.89 \pm 0.07$ for SwinFusion, $0.90 \pm 0.07$ for U2Fusion, and $0.92 \pm 0.04$ for IFCNN. The dice score of MedFusionGAN was significantly (*p*-value $< 0.05$) higher than other listed DL methods. The HD value of MedFusionGAN was statistically significant lower (*p*-value $< 0.05$) than the second best fusion method, SwinFusion, with HD value of $1.69 \pm 0.43$ mm (see Fig. 9d).

## Discussion

We proposed an end-to-end deep learning method, MedFusionGAN, to fuse CT and high-resolution 3D T1-Gd MRI images to generate fuse images containing both CT and MRI contrasts. Our qualitative and quantitative results suggested that the end-to-end unsupervised GAN could transfer MRI soft-tissue contrast and CT bone information to the fused image.

MedFusionGAN was qualitatively and quantitatively compared with 15 state-of-the-art traditional and DL methods. Qualitatively, traditional methods added spatial distortion to the fused image, did not deliver the MRI soft-tissue contrast, or partially located CT bone information (see Fig. 4). DL methods including CNN-Fuse, FusionGAN, and SESF-Fuse did not generated fusion images that combined CT bone structure with MRI soft-tissue contrast. However, MedFusionGAN could combine bone structure from CT and soft-tissue contrast from MRI. FPDE added distortion in the coarse regions that might be attributed to its differential operations. GTF produced edges with considerable differences from the surrounding tissues. However, it did not transfer the soft-tissue contrast of the MRI due to the zero gradient of the MRI soft-tissue. The MedFusionGAN could produce soft-tissue of MRI and more consistently than the traditional and DL fusion methods. The red boxes in Figs. 4 and 5 serve to zoom in on the soft-tissue contrast in the cancerous region and, like the profile along the tumor, illustrates the consistency of our method for delivering soft tissue contrast (see Fig. 8). White boxes in Figs. 4 and 5 illustrate the spatial contrast between scalp and skull where our model generated fusion images with a distinct boundary between the regions. Quantitative metrics demonstrating MedFusionGAN fusion images spatial contrast, edge information, and distortions were calculated and compared with traditional and DL methods (Table 2 and  3). The proposed MedFusionGAN outperformed the state-of-the-art traditional and DL methods in six out of nine quantitative metrics, and, respectively, got the second performance rank in three and two quantitative metrics.

The proposed method was not compared with the previous use of CNN deep learning methods that were primarily proposed for satellite images fusion (infrared and visible images) [23, 25] since they were not end-to-end techniques. Those methods used only one of the source images to train autoencoders to extract features. Then the trained autoencoders on one of the source images were used in the test step to extract features from the source images and were fused using different strategies to combine the features.

Prior research involving GANs [35, 36] predominantly took two approaches: using only one of the source images to train the discriminator or employing two separate discriminators for the two source images. The former approach resulted in a lack of information from one of the source images during discriminator training, causing GANs to generate fusion images that closely resembled the source images (visible images in satellite image fusion scenarios). The latter approach, which utilized two discriminators, posed challenges in achieving a balanced training process to prevent mode collapse. Furthermore, when fusing multiple source images, such as various MRI sequences with CT or positron emission tomography images, the need for m different discriminators added complexity to the training process.

Moreover, the limited dataset size of fewer than 50 image pairs complicates drawing robust conclusions from their results. The comparative CNN models relied on engineered feature fusion methods, which may not generalize effectively for datasets with diverse imaging parameters. Additionally, traditional methods attempted to fuse images using engineered fusion techniques in non-spatial domains, which may not be suitable or robust for datasets with domain shifts.

The MedFusionGAN is a novel deep learning model that fuses 3D T1-GD MRI and CT brain images from multiple imaging centers with different tumor types. This model has been used in an unsupervised framework, which differs from the traditional supervised medical image processing and analysis techniques such as image segmentation, reconstruction [31], and image-to-image translation [30]. Our method was able to outperform the traditional and DL image fusion methods by avoiding any form of mathematical summation while still producing fusion images with better spatial contrast and resolution than other GAN models. Additionally, our algorithm is end-to-end that only requires training one discriminator for stability purposes and leveraging information of the source images compared to other GANs that require more complex training procedures [35, 36].

In summary, the MedFusionGAN provides a powerful tool for medical imaging research due to its ability to accurately fuse various types of brain scans into high quality composite images without requiring extensive manual intervention or time consuming calculations when compared with existing approaches in this field like naive overlay of two images. Our method requires 1.9 second to fuse the source images. This low run-time makes it suitable for online applications. In addition, it can be well integrated with the IGART, which uses the traditional methods with long processing time to fuse the images, with hardly any requirement of the external knowledge. Furthermore, it can be applied across multiple centers regardless of their imaging parameters or tumor type making it applicable for both clinical practice as well as research applications where data needs to be fused quickly yet accurately between different sources in order achieve meaningful results faster than before possible using traditional methods alone.

The novel fusion method presented in this study could have multiple applications for radiation therapy. Three specific applications will be investigated in the future: (1) because fused images maximize the information content of both CT and MRI modalities, we hypothesize that feeding these images to auto-segmentation algorithms could lead to improved performance; (2) the fused images could help treatment planning of GK-SRS that is typically done on MRI-only datasets; (3) performing the fusion of a planning MRI with daily CT or CBCT could help IGART workflow by offering an image that combine the contrast of MRI with the anatomy-of-the-day of a CBCT.

Nonetheless, conventional image fusion methods necessitate source images to be perfectly aligned; otherwise, fusion images may exhibit undesirable artifacts known as stitching ghosts [28]. Achieving precise alignment of medical images can be challenging and is seldom performed in diagnostic settings. In the future study, we will also assess the performance of our model when trained on datasets containing misaligned source images.

## Conclusion

The MedFusionGAN offers an efficient way of fusing multi center brain 3D T1-Gd MRI and CT images along with different tumours. It could fuse the source images with the highest statistical and the highest gradient information of the source images that will improve tumor and OARs delineation compared with other state-of-the-art traditional and DL methods. The increase in contour accuracy would potentially help to lower the needed margins and thus help to reduce side effects and allow for higher prescribed doses. Thus, the radiation treatments including high-dose GK-SRS would be more effective.

### Supplementary Information

**Additional file 1.**

## Data Availability

The brain dataset was obtained from The Cancer Imaging Archieve (https://wiki.cancerimagingarchive.net/pages/viewpage.action?pageId=95224486).

## Footnotes

1 https://fsl.fmrib.ox.ac.uk/fsl/fslwiki/FLIRT

2 https://pytorch.org/.

3 https://www.slicer.org/.
