# Supplementary material for: MedFusionGAN: multimodal medical image fusion using an unsupervised deep generative adversarial network
Source: BMC Med Imaging. 2023 Dec 7;23:203. doi: 10.1186/s12880-023-01160-w (PMC10704723; doi:10.1186/s12880-023-01160-w)
Supplement: Supplementary file 1 — Additional file 1. [file 12880_2023_1160_MOESM1_ESM.pdf]

Supplementary File for:
MedFusionGAN: Multimodal Medical Image Fusion Using an Unsupervised Deep Generative Adversarial Network

Mojtaba Safari[1,2,‡], Ali Fatemi, PhD[3,4], Louis Archambault, PhD[1,2]
[1]Département de physique, de génie physique et d'optique, et Centre de recherche sur le cancer, Université Laval, Québec, Québec, Canada, [2]Service de physique médicale et radioprotection, Centre Intégré de Cancérologie, CHU de Québec – Université Laval et Centre de recherche du CHU de Québec, Québec, Québec, Canada, [3]Department of Physics, Jackson State University, Mississipi, USA [4]Merit Health Central, Department of Radiation Oncology, Gamma Knife Center, Mississipi, USA, [‡]Corresponding Author

Author to whom correspondence should be addressed. email: mojtaba.safari.1@ulaval.ca

I. Quantitative metrics

The brief definition of the metrics are as follows:

1. **Entropy (H):** This metric measured in bits provides the amount of information required to code a given image. The higher the image entropy is, the better the image fusion result is. It is defined as follows:

$$H(X) = -\sum_{x} p(x) \log_2(x) \tag{1}$$

where $p(x)$ is probability image intensity x for a give image X.

2. **Standard deviation (SD):** SD given in Equation 2 is a statistical measure that provides information about image contrast. For a noiseless image, an higher SD means a better image contrast.

$$SD = \sqrt{\mathbb{E}\{(X - \mathbb{E}\{X\})^2\}} \tag{2}$$

3. **Mean gradient (MG):** MG quantifies an image's gradient information, and higher MG means more edge information. MG is mathematically defined as follows:

$$SD = \frac{1}{\Omega} \sum_{x \in X} \sqrt{0.5 \times ((\mathbf{D_1}x)^2 + (\mathbf{D_2}x)^2)} \tag{3}$$

Where $\mathbf{D_i}$ is a differentiation along a given dimension i, and Ω is the image size.

4. **spatial frequency (SF):** SF measures an image gradient (image edge) and texture. It is calculated using row frequency (RF) and column frequency (CF) as follows:

$$SD = \sqrt{RF^2 + CF^2} \tag{4}$$

where $RF = \sqrt{\frac{1}{\Omega}\sum_{x\in X}(\mathbf{D_1}x)^2}$ and $CF = \sqrt{\frac{1}{\Omega}\sum_{x\in X}(\mathbf{D_2}x)^2}$. An image with a higher SF provides more image edge information, and human eyes are more sensitive to images with higher SF.

5. **Mutual information (MI):** For a given two images X and F with marginal probability distributions $p_X(x)$ and $p_F(f)$ and joint probability $p_{X,F}(x, f)$. X and F are statistically independent if $p_{X,F}(x, f) = p_X(x).p_F(f)$. The mutual information $I(X, F)$ of X and F quantify the distance between the joint distribution $p_{X,F}(x, f)$ and the distribution when two distributions are statistically independent $p_X(x).p_F(f)$ using the Kullback-Leibler measure, i.e.,

$$I(X, F) = \sum_{x,f} p_{X,F}(x, f) \log \frac{p_{X,F}(x, f)}{p_X(x).p_F(f)} \tag{5}$$

It is related to the entropy of the images as follows:

$$\begin{aligned} I(X, F) &= H(X) + H(F) - H(X, F) \\ &= H(X) - H(X|F) \\ &= H(F) - H(F|X) \end{aligned} \tag{6}$$

where $H(\bullet)$, $H(\bullet,\bullet)$, and $H(\bullet|\bullet)$ are the entropy (given in Equation 1), joint entropy ($= -\sum_{x,f} p_{X,F}(x, f) \log p_{X,F}(x, f)$), and conditional entropy ($= -\sum_{x,f} p_{X,F}(x, f) \log p_{X|F=f}(x)$), respectively.

6. **Normalized cross correlation (NCC):** NCC measures linear similarity between two images using the direct use of image signal intensities. It is calculated as follows:

$$NCC = \lambda_x.r_{X,F} + \lambda_y.r_{Y,F} \tag{7}$$

$r_{Z,f} = \frac{\mathbb{E}\{(Z-\mathbb{E}\{Z\}).(F-\mathbb{E}\{F\})\}}{\sqrt{\mathbb{E}\{(Z-\mathbb{E}\{Z\})^2\}}.\sqrt{\mathbb{E}\{(F-\mathbb{E}\{F\})^2\}}}$ where $Z \in \{X, Y\}$. It ranges from 0 to 1 and greater value means a higher similarity between source images and fused image.

7. **Peak signal-to-noise ratio (PSNR):** PSNR calculated in decibel measure image representation fidelity. It is a ratio between maximum signal intensity power and the noise distortion power, i.e.,

$$PSNR = 10 \log_{10} \frac{s^2}{MSE} \tag{8}$$

where s is the maximum signal intensity. MSE given in Equation 9 is the mean squared error that measure the image similarity.

$$MSE = \sum_{z \in \{X,Y\}} \lambda_z . \frac{1}{M.N} \sum_{i}^{M} \sum_{j}^{N} (z_{i,j} - F_{i,j})^2 \tag{9}$$

8. $\mathbf{Q^{XY/F}}$: $Q^{XY/F}$ quantifies the amount of the edge information of the fused image. Considering the fusing source images X and Y and fused image F, edge ($g(i,j)$) and orientation ($\alpha(i,j)$) for each pixel is calculated as follows:

$$\begin{aligned} g_Z &= \sqrt{(\mathbf{D_1}Z)^2 + (\mathbf{D_2}Z)^2} \\ \alpha_Z &= \tan^{-1}\left(\frac{(\mathbf{D_2}Z)^2}{(\mathbf{D_1}Z)^2}\right) \end{aligned} \tag{10}$$

where $Z \in X, Y$ and $\mathbf{D_i}Z$ is the differential of Z along dimension i.

The relative edge strength (G^{ZF}) and orientation (A^{ZF}) of an input image Z ($\in \{X,Y\}$) with respect to F are as follows:

$$\begin{aligned} G^{ZF}(i,j) &= \begin{cases} \frac{g_F}{g_Z} & if\ \ g_Z > g_F \\ \frac{g_Z}{g_F} & otherwise \end{cases}, \text{ where } Z \in \{X,Y\} \\ A^{ZF}(i,j) &= \frac{||\alpha_Z(i,j) - \alpha_F(i,j)| - \pi/2|}{\pi/2}, \text{ where } Z \in \{X,Y\} \end{aligned} \tag{11}$$

Then, we can estimate the perceptual information loss in fused image for an input image Z ($\in \{X,Y\}$) as follows:

$$\begin{aligned} Q_g^{ZF} &= \frac{\Gamma_g}{1 + e^{k_g}(G^{ZF} - \sigma_g)} \\ Q_\alpha^{ZF} &= \frac{\Gamma_\alpha}{1 + e^{k_\alpha}(A^{ZF} - \sigma_\alpha)} \end{aligned} \tag{12}$$

where constants Γ_g, k_g, σ_g, Γ_α, k_α, and σ_α determine the exact shape of the sigmoid functions that form the edge strength and orientation values. Equation 13 calculates the edge information preservation.

$$Q^{ZF} = Q_g^{ZF} . Q_\alpha^{ZF} \tag{13}$$

where $Q^{ZF} = 1$ means lossless image fusion.

Finally, we obtain the fusion edge preservation from the weighted sum of the edge information, i.e.,

$$Q^{XY/F} = \frac{\lambda_X Q^{XF} + \lambda_Y Q^{YF}}{\lambda_X + \lambda_Y} \tag{14}$$

$Q^{XY/F} \in [0,1]$ where the higher value means a better image fusion with lower information loss.

9. **Structural similarity index (SSIM):** SSIM assess the structural similarity of two images. It consists of three pixelwise comparisons including luminance, contrast, and structure. SSIM for two images Z and F is defined as follows:

$$SSIM(Z,F) = \frac{(2\mu_Z\mu_F + C_1)(2\sigma_{ZF} + C_2)}{(\mu_Z^2 + \mu_F^2 + C_1)(\sigma_Z^2 + \sigma_F^2 + C_2)} \quad (15)$$

where μ_z, σ_F, and σ_{ZF} are the local mean, local standard deviations, and local covariance between images Z and F, respectively. C_1 and C_2 are the constant parameters to stable the SSIM. The SSIM between source images and fused image is a weighted sum of the Equation 15 and is defined as:

$$SSIM = \lambda_X^{\text{SSIM}}.SSIM(X,F) + \lambda_Y^{\text{SSIM}}.SSIM(Y,F) \quad (16)$$
